# The Treatment of Depressed Chinese Americans Using Qigong in a Health Care Setting: A Pilot Study

**DOI:** 10.1155/2013/168784

**Published:** 2013-04-16

**Authors:** Albert Yeung, Lauren E. Slipp, Jolene Jacquart, Maurizio Fava, John W. Denninger, Herbert Benson, Gregory L. Fricchione

**Affiliations:** ^1^Benson Henry Institute, Massachusetts General Hospital, Boston, MA 02114, USA; ^2^Depression Clinical and Research Program, Massachusetts General Hospital, One Bowdoin Square, 6/F, Boston, MA 02114, USA; ^3^South Cove Community Health Center, Boston, MA 02111, USA

## Abstract

*Background*. This pilot study examined the feasibility and efficacy of providing Qigong treatment in a health center to Chinese Americans with major depressive disorder (MDD). *Methods*. Fourteen Chinese Americans with MDD were enrolled, and they received a 12-week Qigong intervention. The key outcome measurement was the 17-item Hamilton Rating Scale for Depression (HAM-D_17_); the Clinical Global Impressions-Severity (CGI-S) and -Improvement (CGI-I), the Quality of Life Enjoyment and Satisfaction Questionnaire, Short Form (Q-LES-Q-SF), and the Multidimensional Scale of Perceived Social Support (MSPSS) were also administered. Positive response was defined as a decrease of 50% or more on the HAM-D_17_, and remission was defined as HAM-D_17_ ≤ 7. Patients' outcome measurements were compared before and after the Qigong intervention. *Results*. Participants (*N* = 14) were 64% female, with a mean age of 53 (±14). A 71% of participants completed the intervention. The Qigong intervention resulted in a positive treatment-response rate of 60% and a remission rate of 40% and statistically significant improvement, as measured by the HAM-D_17_, CGI-S, CGI-I, Q-LES-Q-SF, and the family support subscale of the MSPSS. *Conclusions*. The Qigong intervention provided at a health care setting for the treatment of primary care patients with MDD is feasible. Further studies with larger sample sizes are warranted.

## 1. Introduction

### 1.1. Depression and Disparities in Treatment among Chinese Americans

There are tremendous racial health disparities in the treatment of depression [1]. Ethnic minorities face both practical and cultural barriers to mental health care. Asian Americans frequently lack the resources to seek help, suffer from language barriers, and hold strong stigma towards psychiatric illnesses, leading to the undertreatment of major depressive disorder (MDD) [2]. There is a pressing need for culturally sanctioned interventions for depressed Asian Americans.

### 1.2. Qigong for the Treatment of Depression

Qigong is a form of exercise that incorporates orchestrated body postures, breath practices, and meditation to attain deeply focused and relaxed states [3]. The Qigong practices are thought to activate naturally occurring physiological and psychological mechanisms of self-repair and health recovery [4]. Most Qigong forms involve slow gentle movements that can be easily adapted and are thus appropriate for people of all ages, fitness levels, and physical conditions. Furthermore, Qigong is relatively inexpensive and easy to learn and requires very little space to practice.

The health effects of Qigong have been reported in various populations with chronic conditions, including high blood pressure [5, 6], bone loss [7], and cardiac disease rehabilitation [8]. While there is increasing evidence on the benefits of the Qigong practice for physical symptoms, its effects on psychological symptoms have yet to be established. Preliminary research has shown Qigong's beneficial effects on a range of psychological well-being measures, including mood, anxiety/depression, general stress management, and quality of life [9–12], but most of the studies recruited healthy subjects or patients with a chronic medical condition but not patients diagnosed with MDD. It has been proposed that Qigong, as a mind-body group exercise, is useful for self-management of depression by alleviating the stress effects on the hypothalamic-pituitary-adrenal axis, decreasing negative thoughts, and increasing social support [13, 14]. 

In this study, we examined the feasibility and safety of offering Qigong at a health center for Chinese Americans with MDD as a stress management technique. We hypothesize that it is feasible and safe to use Qigong for treating Chinese Americans with MDD in a health care setting and that Qigong is effective in reducing depressive symptoms and improving social functioning, based on standardized questionnaires on depression, quality of life, and social support as perceived by the patient. 

## 2. Methods

### 2.1. Participants

 Fourteen Chinese Americans were recruited from the primary care clinics at South Cove Community Health Center (South Cove) between May and October 2011. South Cove is a federally-funded community health center in Boston that serves predominantly Chinese Americans. In 2011, South Cove served more than 25,000 patients, and the majority of them (>92%) were Chinese immigrants. All participants were required to be fluent Chinese speakers both to ensure comprehension in the Qigong classes, which were conducted in Chinese (Cantonese and Mandarin), and to encourage social interaction and mutual support. The study was approved by the Institution Review Board (IRB) of the Massachusetts General Hospital.

 Inclusion criteria included (1) self-identification as being of Chinese ethnicity and fluent in Mandarin and/or Cantonese, (2) 18–70 years of age, (3) Diagnostic and Statistical Manual of Mental Disorders, Fourth Edition (DSM-IV), diagnosis of MDD, and (4) baseline score on the 17-item Hamilton Rating Scale for Depression (HAM-D_17_) ≥ 12. Exclusion criteria included (1) primary psychiatric diagnosis other than MDD, (2) history of psychosis, mania, or severe cluster B personality disorder, (3) being judged by the investigators to have unstable medical conditions, (4) having current active suicidal or self-injurious potential necessitating immediate treatment, and (5) regular practice of Qigong or other forms of mind-body intervention in the past 3 months. Participants receiving treatment for depression, including antidepressants, conventional psychotherapy, or complementary treatments, were allowed to continue with their treatment and were advised not to change their existing treatments during the study. 

### 2.2. Participant Enrollment

 As for recruitment, fliers emphasizing the use of Qigong for stress management were placed at South Cove. This is considered a culturally sensitive way to recruit less acculturated Chinese patients as they tend to have strong stigma against depression or any mental illness. In addition, primary care physicians at South Cove were encouraged to refer patients to this study.

 Potential participants were prescreened over the phone by our bilingual research staff using an IRB-approved protocol. They were then scheduled for a screen visit, where a bilingual investigator consented each participant and conducted interviews. A psychiatrist administered the Chinese bilingual version of the semistructured psychiatric interview (CB-SCID-I/P) [15] to assess the diagnosis of MDD and administered the HAM-D_17_ to determine eligibility. 

### 2.3. Intervention

 The Qigong intervention consisted of 1-hour group classes held twice weekly for 12 weeks in a large conference room in the health center. The instructor had taught Qigong in a community setting for more than 20 years. The instructor followed a standard protocol [16, 17]; the 12 weeks of training started with 20 minutes of traditional warm-up exercises which involved arm swinging; gentle stretches of the neck, shoulders, spine, arms, and legs; and traditional breathing methods. These exercises focus on releasing tension in the body, incorporating mindfulness and imagery into movement, increasing awareness and efficiency of breathing, and promoting overall relaxation of body and mind. After the warm-up exercise, there was a 10-minute break to encourage peer learning and discussion to facilitate social interaction and mutual support, which is considered an important therapeutic element in Qigong. The Qigong exercise instruction (30 minutes) following the intermission included repetitions of slow, standardized Qigong movements. Classes were conducted in Mandarin Chinese. Participants also received a DVD demonstrating the exercises from the instructor and were encouraged to practice at home at least 3 times per week and to record how much they practiced. 

### 2.4. Outcome Assessments

 Outcome measures were assessed at baseline, week 6, and week 12. At each assessment, participants were administered the HAM-D_17_ [18, 19], the Clinical Global Impressions-Severity (CGI-S) and -Improvement (CGI-I) [20], the Quality of Life Enjoyment and Satisfaction Questionnaire, Short Form (Q-LES-Q-SF) [21], and the Multidimensional Scale of Perceived Social Support (MSPSS) [22] which has three subscales: family (MSPSS-FA), friends (MSPSS-FR), and significant others (MSPSS-SO). 

 The HAM-D_17_ is a widely studied instrument for depression, and its reliability and validity are high [23]. The Chinese translated version of the HAM-D has been shown to have adequate reliability and validity in an earlier study [24]. The CGI-S measures the current condition of the patient, as judged by the clinician, on a scale of 1–7 (1 reflecting normal and 7 reflecting the most severely ill patients), and the CGI-I measures the degree of improvement, as judged by the clinician, since the start of treatment on a scale of 1–7 (1 being very much improved and 7 being very much worse). 

The Q-LES-Q-SF is a self-report measure, and each item is rated from 1 (very poor) to 5 (very good). Results are presented as the total score and as an average of the single overall assessment item, with higher scores representing better quality of life. The Chinese version of the Q-LES-Q-SF has been demonstrated to have good reliability and validity among Chinese patients with psychiatric disorders [25]. The MSPSS is a self-administered 12-item scale used to assess perceptions of social support from family members, friends, and significant others. Items are rated on a 7-point Likert scale (1: very strongly disagree; 7: very strongly agree), with higher scores indicating greater level of perceived support. Confirmatory factor analysis has consistently reported a 3-factor solution: family (MSPSS-FA), friends (MSPSS-FR), and significant others (MSPSS-SO) [26]. Internal consistency of the Chinese version is good [27, 28].

 In addition, patients were asked to fill out the “Beliefs and Expectations of Qigong” [29], a 4-point Likert scale (0 = no, 1 = maybe, 2 = yes, and 3 = definitely) for the participants to report their opinions on how effective they consider Qigong as treatment of depression. At each class, participants were asked to fill in an attendance sheet, an adverse events log, and an adherence to the Qigong practice log to report the frequency and duration of their Qigong practice in the past week. 

### 2.5. Data Analyses

All the participants were evaluated at week 6 and week 12 assessments; measurements made at the baseline were compared to week 12 assessments. Descriptive statistics were applied to the demographic and clinical characteristics of all the participants. Outcome analyses were restricted to completers of the intervention, defined as those who have attended more than 15 (62.5%) of the 24 training sessions. Positive response to treatment was defined as a decrease of 50% or more of a patient's HAM-D_17_ score, and remission was defined as having a score of 7 or less on the HAM-D_17_ at the last assessment. Participants' HAM-D_17_ scores, CGI-S, and Q-LES-Q-SF measurements before and after the intervention were compared using the nonparametric Wilcoxon signed-rank test, in view of the small sample size. For CGI-I analysis at week 12, we used one-sample *t*-test comparing the mean against the hypothesized value of zero for no change. Statistical analyses were conducted using SPSS software, version 17.0. For all analyses, significance was set at the 0.05 alpha level.

## 3. Results

 Fourteen Chinese Americans with MDD were enrolled in the study (64% female, mean age 53 ± 14). The baseline and demographic characteristics of participants are listed in Table 1. Most participants had positive expectations that Qigong would help their depression (“not helpful”: 0%, “maybe”: 64%, “yes”: 29%, and “definitely”: 7%). Ten (71%) participants completed the intervention, based on having attended more than 15 (62.5%) sessions. No adverse events due to the Qigong intervention were reported. The participants showed a lot of enthusiasm in class. During the 10-minute intermission time, they were eager to share learning experience with their peer participants and asked the instructor questions on the theories and technical aspects of Qigong. Participants reported practicing Qigong on the average four days a week at home (4.0 ± 2.0) both at week 6 and week 12 assessments, which exceeded our expectation of three days a week of home practice. 

At baseline, patients had an average HAM-D_17_ score of 21.4, which corresponded to being moderately depressed [18]. The Qigong intervention resulted in a positive treatment-response rate of 60% and a remission rate of 40%, and statistically significant improvement in HAM-D_17_ (from 21.4 ± 3.0 to 9.4 ± 5.3, *z* = −3.3, and *P* = 0.001) CGI-S (from 4.4 ± .5 to 2.7 ± 1.1, *z* = −2.8, and *P* = .004), CGI-I (mean of 2.1 ± 1.1, *t* = −6.7, df = 13, and *P* = .00), Q-LES-Q-SF (from .4 ± .1 to .6 ± .1, *z* = −3, and *P* = .003), and MSPSS-FA (from 19.0 ± 5.3 to 20.7 ± 5.3, *z* = −2.1, and *P* = .036). Both the MSPSS-SO and MSPSS-FR showed improvement after the intervention, but the changes were not statistically significant. The results are shown in Table 2.

## 4. Discussion

This pilot study provides preliminary information on the potential impact of a Chinese traditional healing practice to treat MDD in underserved Chinese Americans, who historically underutilize conventional psychiatric treatments. If the efficacy of Qigong in MDD can be shown, it has the potential to significantly impact a large proportion of underserved ethnic minorities with mental health issues. This study demonstrates the feasibility and safety of using Qigong as an intervention for Chinese Americans with MDD. We encountered little difficulty in recruiting for this study, and the Chinese community responded positively to the idea of using Qigong as an intervention for depression. At baseline evaluation, all participants had positive expectations of Qigong. A 71% of participants in the Qigong group completed the intervention (>62.5% attendance) demonstrating satisfactory compliance to treatment. The completers reported that they practiced Qigong four times a week at home in addition to going to the class twice weekly. Qigong also appeared safe in this population, given that we observed no adverse events. This is consistent with findings from previous clinical trials [5, 6]. The response and remission rates after the Qigong intervention were satisfactory even compared to the results from antidepressant treatment as reported from the STAR-D study [30], the largest clinical trial on MDD in the USA. 

The offering of Qigong as an intervention in a health care setting is an innovative approach. Despite the fact that Wagner (1998) pointed out the importance of the use of a multifaceted approach, including exercise, relaxation training, and self-management for treatment of chronic disease, patients are generally left on their own to look for these resources in the community [31]. For the underserved minorities, such resources are not readily available to them. 

In this study, we demonstrated the feasibility with preliminary data on the effectiveness of offering Qigong within a community health center to Chinese immigrants with MDD. Placing self-management trainings within a health center makes it more convenient for patients and is a step to increase integration of medical and behavioral treatment. Qigong has been shown to have beneficial effects for both physical and psychological conditions [32, 33]. Further studies should examine whether such a model would be efficacious as a therapeutic intervention and/or preventive measure for depression and other chronic diseases. 

 We would like to acknowledge the following limitations of this pilot study. First, this is an open label study with a small sample size and no control group in which all subjects received the intervention. While we found significant improvements in many outcome variables, the absence of a control group obviates the possibility of stating definite conclusions regarding the effectiveness of Qigong for treating depression. Patients who were taking antidepressant medications may improve because of medication effects, but only 2 (14%) subjects were on antidepressant medications. It is possible that patients' symptomatic improvement was due to the passage of time. This could happen if patients sought help while they were highly symptomatic as depressive symptom severity may fluctuate and decrease over time. Second, it is unclear if patients' improvement in the intervention group was a result of Qigong or from social interaction from participating in the study, the establishment of a new structure in life, or being stimulated to engage in a new commitment to learn and practice Qigong. Future attention-controlled and mechanistic studies might further investigate the differential impact of Qigong and of social interaction. Another limitation is the issue of generalizability. As patients in this study were predominantly recent Chinese immigrants, we cannot be sure whether these results would generalize to other populations. Further studies will be needed to examine if Qigong is effective for treating depression in the mainstream population and in other ethnic minority groups. 

## 5. Conclusions

Qigong as a stress reduction intervention in a health care setting is safe, feasible, and well received. A 12-week Qigong intervention may be effective for improving symptoms and inducing remission in Chinese Americans with MDD. Future studies with larger sample sizes randomized to the intervention and control groups and the inclusion of biological outcome measures will be needed to provide more definitive outcomes.

## Figures and Tables

**Table 1:** Baseline characteristics of study participants (*n* = 14).

Characteristics	% (*n*)	Mean	(SD)
Age (years)		53	(14)
Gender (female)	64 (9)		
Marital status (married)	71 (10)		
Education (years)		10.4	(1)
Employment status (employed)	57 (8)		
Currently receiving antidepressants	14 (2)		
Beliefs on usefulness of Qigong on depression			
Not helpful	0 (0)		
May be helpful	64 (9)		
Yes	29 (4)		
Definitely helpful	7 (1)		
HAM-D_17_ (baseline)		21.4	(3)
CGI-S (baseline)		4.4	(.5)
Q-LES-Q score		.4	(.1)
MSPSS-SO (significant other)		16	(6)
MSPSS-FA (family)		19	(5)
MSPSS-FR (friends)		15	(5)

**Table 2:** Depression treatment outcomes in completers of the Qigong intervention (*n* = 10).

Treatment outcomes	Initial assessment		Final assessment			*z* or *t *value*	*P* value
	Mean	(SD)	% (*n*)	Mean	(SD)		
Response rate			60 (6)				
Remission rate			40 (4)				
CGI-S	4.4	(0.5)		2.7	(1.1)	*z* = −2.8	.004**
CGI-I (lower scores reflect more improvement)				−2.1	(1.1)	*t* = 6.8 (df = 13)	.00*
HAM-D_17_	21.8	(2.8)		9.4	(5.3)	*z* = −3.3	.001**
Q-LES-Q-SF	0.4	(0.1)		0.6	(0.1)	*z* = 3.0	.003**
MSPSS-SO	15.5	(5.6)		18.6	(5.9)	*z* = −1.7	.085
MSPSS-FA	18.9	(5.3)		20.7	(5.3)	*z* = −2.1	.036**
MSPSS-FR	14.9	(5.3)		17.4	(5.0)	*z* = −1.5	.13

*Based on Wilcoxon signed-rank test or one-sample *t*-test.

**Statistically significant (*P* < 0.05).
